# Successful therapies for Alzheimer’s disease: why so many in animal models and none in humans?

**DOI:** 10.3389/fphar.2014.00146

**Published:** 2014-06-25

**Authors:** Rafael Franco, Angel Cedazo-Minguez

**Affiliations:** ^1^Division of Neurosciences, Centro de Investigación Médica Aplicada, Universidad de NavarraPamplona, Spain; ^2^Department of Biochemistry and Molecular Biology, Faculty of Biology, Universitat de BarcelonaBarcelona, Spain; ^3^Department of Neurobiology, Care Sciences and Society, Center for Alzheimer Research, Karolinska InstitutetHuddinge, Sweden

**Keywords:** adrenergic receptors, lost-in-translation research, resveratrol, transgenic AD models, biomarkers

## Abstract

Peering into the field of Alzheimer’s disease (AD), the outsider realizes that many of the therapeutic strategies tested (in animal models) have been successful. One also may notice that there is a deficit in translational research, i.e., to take a successful drug in mice and translate it to the patient. Efforts are still focused on novel projects to expand the therapeutic arsenal to “cure mice.” Scientific reasons behind so many successful strategies are not obvious. This article aims to review the current approaches to combat AD and to open a debate on common mechanisms of cognitive enhancement and neuroprotection. In short, either the rodent models are not good and should be discontinued, or we should extract the most useful information from those models. An example of a question that may be debated for the advancement in AD therapy is: In addition to reducing amyloid and tau pathologies, would it be necessary to boost synaptic strength and cognition? The debate could provide clues to turn around the current negative output in generating effective drugs for patients. Furthermore, discovery of biomarkers in human body fluids, and a clear distinction between cognitive enhancers and disease modifying strategies, should be instrumental for advancing in anti-AD drug discovery.

## INTRODUCTION

**FIGURE 1:**
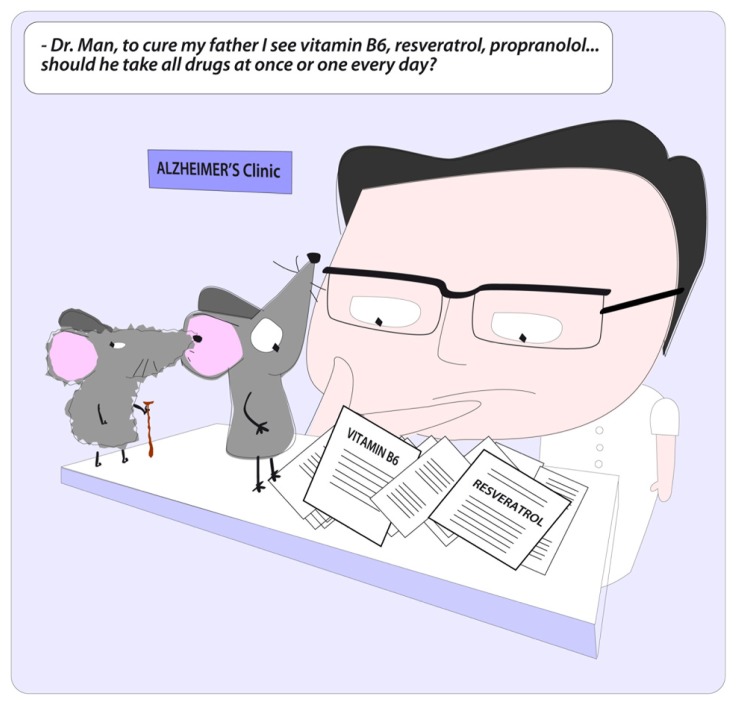
**Cartoonial metaphor of the success in relieving the cognitive deficits of AD mouse models.** A variety of different compounds are proven successful as cognitive enhancers and neuroprotetive in transgenic AD models. Copyright retained by Nuria Franco (franconuria@gmail.com).

In humans, there are only two types of drugs approved for Alzheimer’s disease (AD) that unfortunately are not of much relief: acetyl cholinesterase inhibitors and modulators of *N*-methyl-D-aspartate (NMDA) receptors. In animals, however, every other treatment tested has been successful. The vast amount of effort devoted to find a cure or a relief to AD patients, i.e., to translate pre-clinical findings to the bedside, has been to date fruitless. It is about time to covert the feeling of lost-in-translation research into profitable knowledge. Even if transgenics were good models for human AD, it is intriguing that many different compounds targeting different pathways enhance cognition (see **Figure 1**). Careful analysis of why different approaches reduce symptoms and/or degeneration in animals will surely help in accelerating translational research and provide human-successful medications to fight the two sides of AD: neurodegeneration and cognitive dysfunction. Better animal models and good biomarkers for patient subgrouping will be also instrumental for quick advancement in the field. The paper revises all these aspects with the aim to generate debate among scientists.

## ALZHEIMER’S DISEASE FEATURES

One of the distinguishing features of AD pathology is the deposition of Aβ-containing senile plaques and phospho-tau-containing tangles in brain. The discovery of early-onset familial AD (FAD) cases more than 20 years ago identified three relevant genes [amyloid precursor protein (APP) and presenilins 1 and 2, reviewed in Tanzi et al., 1996]. Mutations in these genes cause an overproduction of beta-amyloid (Aβ) or of the longer Aβ peptides. These genetic-based observations, together with the fact that individuals with Down’s syndrome ^1^ show AD-like pathology in their brain, have led the “*amyloid cascade hypothesis*” (Hardy and Higgins, 1992) by which Aβ initiates a chain of events leading to neuroinflammation, neurodegeneration, and clinical manifestations (memory loss and senile dementia). The *amyloid cascade hypothesis* for AD has dominated the thoughts about the etiology and pathogenesis of this disease, and guided the efforts to find treatments. It has been proposed that longer Aβ forms, i.e., amyloid peptides 1–42 or 1–43, are triggering factors for AD (Sandebring et al., 2013). However, the differential toxicities of Aβ forms have not been sufficiently addressed and alternative hypotheses point to either fibrils (Lorenzo and Yankner, 1994), oligomers (Lambert et al., 1998), dimers (Shankar et al., 2008), or soluble forms (Cleary et al., 2005) as the culprit. Similarly, the relative abundance of the different Aβ species or conformations has not been determined in either FAD, sporadic AD or in Down’s syndrome. Still, we have a relatively good understanding of the mechanisms by which these rare genetic mutations lead to AD. However, the precipitating factors that lead to the accumulation of Aβ and also of hyperphosphorylated tau in the much more common sporadic forms of AD are still unknown. As of yet, there is no clear link between Aβ and tau pathologies, as animals carrying FAD genes express huge amounts of amyloid but show no tangle pathology. Also, a major unmet scientific need in the AD field is to understand the normal biochemistry and biological function(s) of APP and its metabolites. Accordingly, the potential risks of targeting Aβ production (in the brain and the periphery) are yet undetermined. Although the reported pathological effects of Aβ are numerous (to date, 2633 articles for “Aβ toxicity” are in Pubmed), senile plaques and Aβ were even looked as protective adaptations to AD (Lee et al., 2004). In parallel to the toxic effects in a variety of cell and animal models, Aβ may activate kinases (Vestling et al., 1999; Cedazo-Minguez and Cowburn, 2001; Tabaton et al., 2010), act as antioxidant (Zou et al., 2002), have anti-microbial activity (Soscia et al., 2010), or modulate cholesterol transport (Yao and Papadopoulos, 2002). As β- (BACE1) and γ-secretases are responsible for Aβ generation, efforts have been made to develop inhibitors for clinical use in AD. However, increasing number of studies reveals their role in the metabolism of multiple substrates, which is a challenge to reach selectivity to only inhibit Aβ production. Moreover, some substrates for these proteases are important for neuronal cell biology. For example, BACE1 cleaves β subunits of voltage-gated sodium channels (Gersbacher et al., 2010), and neuregulins, which are crucial molecules for development and myelinization (Hu et al., 2006; Willem et al., 2006). The same holds true for γ-secretase, a promiscuous di-aspartyl protease responsible for the cleavage of diverse, type I transmembrane proteins. To date, more than 90 proteins have been identified as substrates of this enzyme (Haapasalo and Kovacs, 2011). γ-secretase substrates have different structure, localization, and regulate a wide variety of cellular events, such as cell fate determination, adhesion, migration, neurite outgrowth, axon guidance, or formation and maintenance of synapses (Jurisch-Yaksi et al., 2013). Apart from APP, the most studied γ-secretase substrate is Notch, a signaling molecule critically required for development and cell fate determination. Druggability of γ-secretase has not been an issue, but selectivity to inhibit only APP cleavage is a major problem. In addition to decreasing Aβ, γ-secretase inhibitors affect many proteins inhibiting production of functionally important C-terminal peptides, an outcome with potentially toxic consequences. Therefore, new strategies are needed to develop agents selectively inhibiting cleavage of APP without affecting other substrates. These efforts were propelled by recent discoveries of modulators that control the γ-secretase cleavage of specific substrates by binding and recruiting them to γ-secretase for processing (Barthet et al., 2012).

The APP primary structure strongly suggests that its normal function relates to cell–cell interaction and cell–substrate adhesion processes consistent with a role in development. Furthermore, APP involvement has been reported in cell migration (Rice et al., 2012), trafficking and signaling (Aydin et al., 2012), neuronal calcium homeostasis and synaptic transmission (Octave et al., 2013), and iron-mediated neurotrophic properties (Duce et al., 2010). Targeting the metabolism of APP to reduce Aβ production will also affect the production of other APP metabolites (i.e., sAPP or the amyloid precursor protein intracellular domain, AICD). AICD has more than 20 interacting protein partners, which connect APP to different intracellular signaling pathways including regulation of transcription, apoptosis, and cytoskeletal dynamics (Cowburn et al., 2007; Pardossi-Piquard and Checler, 2012).

## ANTI-AMYLOID AND ANTI-PHOSPHO-TAU APPROACHES

Studies in animal AD models have suggested that removal of Aβ and/or decreasing its production are good strategies for anti-AD therapy. Unfortunately, the numerous clinical trials with agents targeting amyloid have failed to reach the primary clinical endpoints. In the last 3/4 years, four additional clinical trials along this line (inhibition of γ-secretase by semagacestat and avagacestat, and two different anti-Aβ antibodies, bapineuzumab and solanezumab) gave ambivalent results with no obvious positive findings. At present, the AD field is considering whether persevere with Aβ targeting or concentrate efforts and resources on other approaches.

On-going studies such as the Alzheimer’s Prevention Initiative (API) and the Dominantly Inherited Alzheimer Network (DIAN) will determine if clearing Aβ from the brain is effective to treat AD. API is an international public–private consortium established to conduct research on an extended family of 5000 members in Antioquia, Colombia (the world’s largest in which a gene for FAD has been identified). DIAN is an international initiative funded by the US National Institute on Aging (NIA) tracking participants from families in which Alzheimer’s-causing mutations have been identified. Targeting Aβ may be only successful for the early-onset autosomal dominant types of the disease, where increased Aβ production occurs from birth. For the majority of AD cases, where amyloid accumulation is a later event resulting from unknown causes, the strategy is probably not so straightforward.

Despite intraneuronal tangles constituted by abnormally hyperphosphorylated tau are in direct correlation with neuronal death and AD progression, they have historically been second players both to explain cognitive impairment and to be considered in therapy. In contrast to the APP gene, mutations in tau do not cause AD, but fronto-temporal dementia (FTD; Goedert and Jakes, 2005). Tau pathology is present not only in AD but also in several neurodegenerative disorders, pointing out to a central role in the neuronal death mechanisms. In contrast to Aβ, the biological function of tau is well known. Tau regulates microtubule assembly, dynamics, and spatial organization, it and participates in the axonal transport of organelles and vesicles (Ebneth et al., 1998). Tau-induced neurodegeneration is likely a consequence of a loss of biological function together with the initiation of toxic events. There is a strong correlation between tau phosphorylation and tau pathology, and this has led to the rationale of developing tau kinase inhibitors as potential therapeutic agents (Medina and Avila, 2010). Since there is not a single but multiple kinases involved in generating hyperphosphorylated tau *in vivo*, there is a debate about the potential efficacy of targeting the different kinases (Schneider and Mandelkow, 2008). Tau anti-aggregants and tau-based immunotherapy are also being tested but so far without success. For instance, the GSK3β inhibitor tideglusib, tested in a 26-week Phase IIb trial for the treatment of *circa* 300 mild-to-moderate AD patients, failed to meet the primary cognitive endpoint and two secondary endpoints (Medina and Avila, 2014).

As an alternative to kinase inhibition, activation of phosphatases has also been proposed as a strategy for reducing tau phosphorylation. Protein phosphatase 2A (PP2A), the main brain phosphatase, has received special attention. Treatment of tau transgenic mice with the PP2A activator sodium selenate reduced tau hyperphosphorylation and tangle formation, as well as improved memory and prevented neurodegeneration (Corcoran et al., 2010). As PP2A has a broad substrate specificity, allosteric activation of this enzyme to specifically reduce tau phosphorylation is a big challenge. Several other anti-tau treatments were effective in preventing and intervening the progress of tau hyperphosphorylation in animal models, improving neuron function or cognition, for example epithilone D (EpoD; Brunden et al., 2010) or microtubule-stabilizing agents as davunetide (Matsuoka et al., 2008). Disappointingly, a 12-week, placebo-controlled study with intranasal davunetide was recently reported failing to detect a statistically significant benefit in 144 subjects with amnestic mild cognitive impairment (Morimoto et al., 2013).

As in the Aβ field, increasing efforts are being made to design an effective vaccine against tau pathology. Few studies regarding passive immunization against tau protein are currently available (Panza et al., 2012). Also, several studies propose that active immunization may be effective against tau in animal models (Medina and Avila, 2014). Very recently, the first in-man active anti-tau immunization studies have started (AADvac1; http://clinicaltrials.gov/ct2/show/NCT01850238). Similarly to the Aβ-based therapies, a number of key questions remain to be answered in the tau-based immunotherapeutic approaches. Still, we do not know which is the exact species to be targeted (aggregation states, fragments, subtypes), or the mechanism of action by which antibodies clear target molecules. The coming years will tell if current anti-tau immunotherapeutic approaches are effective or if they will be “lost in translation,” as in previous immunization-based strategies.

Methylthioninium chloride (or methylene blue) was presented in 2008 at the International Conference on Alzheimer’s Disease (ICAD) held in Chicago. Methylene blue Phase II clinical trial data suggested that it slowed decline in people with AD by roughly 4-points on the ADAS-Cog. However, methylene blue colors the urine green, and pigments the eyes blue, which raises the question of how the study could be performed blindly. A new version of methylene blue is now heading toward phase 3 testing, though in FTD. The mechanism of action of this compound is unknown, but it has been speculated that it could act as anti-tau aggregation agent. Some studies theorize that the compound blocks aggregation of not only tau but also several other proteins such as α-synuclein, TDP-43, or even Aβ (Sontag et al., 2012), whereas other studies find the compound to be ineffective (Van Bebber et al., 2010). Other suggestions for methylene blue mode of action were autophagy stimulation (Congdon et al., 2012) or enhancement of proteasome activity (Medina et al., 2011).

## THE DIABETES AND VASCULAR LINK TO AD

**FIGURE 2:**
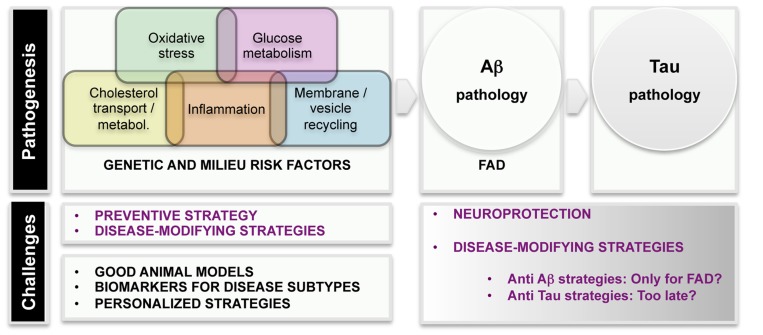
**Scheme of etiological factors and challenges in AD research.** Pathogenesis: epidemiological and genetic findings uncovered several major mechanisms leading to AD pathogenesis. Main AD pathological hallmarks are deposition of Aβ and p-tau in the brain. It is unquestionable that AD starts with Aß pathogenesis in familial AD (FAD) cases. The current knowledge suggests that tau pathology is a later event in the disease.

Even before FAD mutations were detected, epidemiological studies revealed several risk and protective factors for dementia. The development of improved population studies over the years has characterized with great detail which environmental factors confer risk or protection, thus opening the possibility of designing preventive strategies. Several vascular, lifestyle, and genetic risk factors have been recognized and they may act both independently and by potentiating each other (Kivipelto et al., 2008). Epidemiological and biological evidences strongly underlined the importance of the vascular component in AD pathology, (Ligthart et al., 2010) and diabetes, hypertension, and high blood cholesterol levels have been shown to enhance the risk for AD (Imtiaz et al., 2014). In addition to those, other conditions prone to converge into AD have been identified, among them traumatic brain injury (Shively et al., 2012), ischemia/hypoxia (Zhang and Le, 2010), neuroinflammation (Ferreira et al., 2014), and metabolic abnormalities coursing with decreased brain glucose uptake (Gong et al., 2006). **Figure 2** displays a list of etiological factors in human AD.

Based on these risk indicators, the possibility of predicting who will develop AD is very modest; however, the relevance of these correlations lies in that they give clues about pathways/processes leading to AD. Some of those pathways have been somehow confirmed by genome-wide association studies (GWAS). Over the last three to five years, GWAS (with continuous pooling of larger number of samples) have identified common loci (typically frequencies of 10–50%) which have modest effects on risk (with odds ratios in the 1.1–2.0 range; Hardy et al., 2014). In broad terms, GWAS identified cholesterol metabolism, innate immune system, and endosomal vesicle recycling as important contributors to AD. As some of the APP processing occurs in endosomal compartments, proteins participating in vesicle traffic (SORL1, PICALM, and BIN1) are worth being further characterized (Hardy et al., 2014). Very little is known about the therapeutical implications of the discovery but it seems plausible that impairment of vesicle recycling would have detrimental consequences in secretory and/or autophagy pathways.

Before these recent genetic studies, a large amount of evidence suggested a pathogenic link between disruptions in cholesterol metabolism and AD. The strongest known genetic risk factor for sporadic AD is the presence of the E4 allele of the apolipoprotein E (ApoE), which was already detected in 1993 (Corder et al., 1993). ApoE is the major carrier of cholesterol in the central nervous system (CNS) and compared to other apoE isoform carriers, individuals with one or two copies of apoE4 have a higher risk to develop AD. Since the discovery of ApoE4 as a major risk factor for AD, considerable efforts have been made in linking this molecule to Aβ metabolism, aggregation, and deposition. An increased plaque deposition has been observed in apoE4 individuals and in animal models of brain amyloidosis carrying the human apoE4 (Schmechel et al., 1993). ApoE4 has been shown to potentiate Aβ toxicity *in vitro*(Cedazo-Minguez et al., 2001; Manelli et al., 2007) and in animal models (Bales et al., 1999). Studies in microglia have shown that ApoE3 cells can degrade Aβ more efficiently than ApoE4 cells. Moreover, ApoE4 binds Aβ with lower affinity than ApoE3, suggesting that ApoE4 might be less efficient in mediating Aβ clearance (LaDu et al., 1994). On the other hand, the contribution of ApoE4 to tau hyperphosphorylation remains poorly understood.

A reduced capacity for neuronal delivery of cholesterol of apoE4 is believed to have consequences for synaptogenesis and repair mechanisms, and it is likely to directly contribute to disease progression. This notion is supported by GWAS, where in addition to apoE, several other candidate genes related to cholesterol synthesis, transport, uptake, or metabolism were identified (i.e., ABCA7, ABCA1, CLU, CYP46A1; Hardy et al., 2014). New efforts are necessary to understand the underlying mechanisms, as well as to discern if regulation of brain cholesterol metabolism has therapeutic potential.

Neuropathological analysis of AD brains has implicated the complement cascade in relation to AD pathogenesis. Now, genetic analysis has clearly shown that variability in innate immunity is an important determinant of AD risk with several loci pointing to this system. Inflammation has been proposed as an early pathogenic event in the disease (Wilcock and Griffin, 2013). Elevated levels of complement factors in cerebrospinal fluid (CSF) and microglial activation markers have been described in AD brains, but longitudinal data that classify these alterations in relation to onset of amyloid deposition and tau phosphorylation are lacking. Since inflammatory responses can have both beneficial and detrimental effects on the brain (Rivest, 2009), it would be instrumental to delineate ways to regulate neuroinflammation.

We have considerable information on different pathways contributing to the disease. Together, epidemiological and genetic screenings of AD individuals have categorized insulin resistance, deficits in cholesterol transport, hypertension, and neuroinflammation as potential factors in AD physiopathology. One of the challenges for the future would be to discern the overlapping, intersecting or synergic mechanisms leading to brain Aβ accumulation, neuronal tau hyperphosphorylation and synaptic deficits. The existence of variant pathways to AD is probably reflecting the heterogeneous etiology of the disease. It is likely that alteration in different pathways would result in various patient subgroups, which should be treated individually and differentially recruited in clinical trials. The identification of patient subtypes, with homogenous etiology and prognosis, will result in more accurate and personalized treatments. Intensifying innovative basic research will also result in the identification of novel biomarkers for subtyping AD, which is crucial for accurate diagnosis and medicinal interventions.

## MULTIPLE WAYS OF “CURING” ANIMAL AD MODELS

Apart from those affecting Aβ production/processing or those reducing hyperphosporlated tau, a variety of drugs with different modes of action were proved to be efficacious in AD models (**Table 1**). The battery of effective compounds in animal models for AD is continuously increasing. As example, the last two 2013 volumes of the specialized journal, *Journal of Alzheimer’s Disease* have been reviewed (volumes 36 and 37). The main message from selected articles – including literal sentences – is provided below. Carvajal et al. (2013) show that tetrahydrohyperforin, a semi-synthetic derivative of the phytochemical hyperforin, decreases cholinergic markers associated with Aβ plaques and caspase-3 activation in AβPP/PS1 mice. This phytochemical is found in St. John’s wort (*Hypericum perforatum*), which has been considered herbal medicine since the ancient Greeks (Klemow et al., 2011) and is apparently helpful as antidepressant, antibacterial, antiviral, antineoplastic, anti-inflammatory, antioxidant, and neuroprotective (Dinamarca et al., 2006; Griffith et al., 2010). As a further AD-related benefit, the compound induces mitochondrial dynamics and prevents mitochondrial Ca^2+^ overload after Aβ and Aβ–AChE complex challenge in rat hippocampal neurons (Zolezzi et al., 2013). Cheng et al. (2013) show that nicotinamide mononucleotide adenylyltransferase 2 affects tau phosphorylation by regulating the activity of protein phosphatase PP2A, and suggest that this enzyme may serve as a potential target in arresting AD-like tau pathologies. Mitochondria-targeted plastoquinone antioxidant SkQ1 prevents Aβ-induced impairment of long-term potentiation in rat hippocampal slices. Authors summarize that “*in vivo* and *in vitro injection of SkQ1 compensates for Aβ-induced oxidative damage of long-term synaptic plasticity in the hippocampus, which is considered to be the main reason of memory loss and impairment of other cognitive functions associated with AD.*” According to the authors, “*SkQ1 may be considered as a promising candidate for the treatment of early-stage Alzheimer’s disease*.” Olazarán et al. (2013) show that in advanced AD patients, brain stimulation with radio electric asymmetric conveyer technology may improve motor deterioration. Previously, Mannu et al. (2011) had demonstrated that radio electric asymmetric brain stimulation improves behavioral and psychiatric symptoms in AD. By measuring Aβ induction of inducible nitric oxide synthase, Dursun et al. (2013) suggest a crosstalk between Aβ pathology and vitamin D receptor and that vitamin D supplementation should be considered in both treatment and prevention of AD. Veszelka et al. (2013) show that “*docosahexaenoic acid can protect not only neurons but also the other elements of the neurovascular unit from the toxic effects of Aβ42 and this effect may be beneficial in AD*.” Saydoff et al. (2013) report that “*a uridine prodrug improves memory in Tg2576 and in the double mutant Tg2576 x P301L (point mutation in tau) mice and reduces pathological factors associated with AD in related models*.”

**Table 1:** Non-exhaustive list of interventions that have proved efficacious in animal AD models.

Intervention	Comments	Results from tests in humans/patients	Review(s)
Anti-amyloid	Decrease the number of plaques by a variety of strategies: preventing amyloid production, by using antibodies against amyloid or using a vaccine strategy	Yes Negative results	Castellani and Smith (2011)
Anti-ptau	Decrease hyperphosphorylation of tau	Yes Negative results	Fuentes and Catalan (2011)
Anti-diabetic	Hyperglycemia is a risk factor	Yes Inconclusive results	De la Monte and Tong (2014),Ryan et al. (2014),Yang and Song (2013)
Anti-inflammatories	Inflammation is an underlying factor in AD	Yes Incosistent/negative results	Calzà et al. (2013)
Hypocholesterolemics	Hypercholesterolemia is a risk factor	No	Shinohara et al. (2014)
Antioxidants	Elimination of radicals is neuroprotective	Yes Negative results	Rosini et al. (2013)
Dual cholinergic and serotonergic drugs	Synergy of cholinergic tone and anti-depressant action of serotonin	No	Toda et al. (2010)
Epigenetic	Increase epigenetic marks leading to increase in transcription of genes related to memory	No	Cuadrado-Tejedor et al. (2011)
Phosphodiesterase inhibitors	Increase cGMP, activation of CREB and transcription of genes related to memory	No	García-Osta et al. (2012)
Antihypertensives	Hypertension is a risk factor	Yes Results not yet available	Wiesmann et al. (2013b)
Anti-depressant	Not clear if to improve cognition or to address a side symptom in patients	Yes Inconclusive results	Sepehry et al. (2012)
Vitamins and phospholipids	Increased intake of metabolic and coenzyme precursors good to improve cognition	Yes Positive results being revisited (see text)	Engelborghs et al. (2014)
Resveratrol	Phenolic compounds in wine are neuroprotective	No	Davinelli et al. (2012),Virmani et al. (2013)


*An exhaustive summary of all interventions proved successful in animal AD models is out of the scope of the present article.*

Special attention merits the report by Wiesmann et al. (2013a) on improved spatial learning strategy and memory in aged Alzheimer AβPPswe/PS1dE9 mice on a multinutrient diet, as this diet is similar to that in a commercial product that was about to be approved for use in humans. As of March 2014, the www.souvenaid-us.com site indicates that this product is intended for human use:

“… *is a unique nutritional approach providing key nutrients to help support memory in aging adults” and “… contains FortasynConnect, a patented combination of nutrients which includes omega-3 fatty acids, choline, uridine monophosphate and a mixture of antioxidants and B vitamins. The product is not yet available for purchase as it is being further researched and developed*.”

Zhao et al. (2013) reported that “*an early intervention with an estrogen receptor β-selective phytoestrogenic mixture, referred to as the (phyto-β-SERM) formulation, which exhibits 83-fold higher binding selectivity for the estrogen receptor subtype β (ERβ) than for ERα, prolongs survival, improves spatial recognition memory, and slows progression of amyloid pathology in a female AD mouse model*.”

These papers represent a sample from just 6 months of a single journal; they reflect the enormous scientific task devoted to AD in hundred of laboratories around the world, and support the view that targeting different proteins in different pathways is successful when using transgenic AD mouse models

It is worth noting two epidemiological studies in these two volumes of the *Journal of Alzheimer’s Disease*. In a cross-sectional and longitudinal study, Qiu et al. (2013) reported that “*angiotensin converting enzyme inhibitors, especially peripherally acting ones, are associated with a reduced risk of AD in the absence of apoE4, but had no such effect in those carrying the apoE4 allele*.” This paper is noticeable because it is an example of the various papers showing, also in AD mouse models, that peripheral interventions may result in CNS benefits. Even maternal breastfeeding has been analyzed among a cohort of elderly British women to find that “*those who breastfed had lower AD risk than women who did not breastfeed*” (Fox et al., 2013).

## THE GAP BETWEEN MOUSE MODELS AND HUMAN PATIENTS

The reasons for the poor translation of pre-clinical into clinically successful interventions to combat the disease are unknown. The lack of good predictive animal models, good biomarkers for disease progression, and well-defined target populations in clinical trials are strong barriers for demonstrating potential anti-AD benefits. A list of pros and cons of using animal models and patients (**Table 2**) reflects key differences that explain, in part, the difficulties in translating pre-clinical findings to patients.

**Table 2:** Differences between AD models and human AD.

AD animal models		Patients	
Pros	Cons	Pros	Cons
Good for pre-clinical assays	They overexpress mutant versions of human proteins; therefore, they are not suitable models for the most common form of AD (late onset)	They display the real pathology	Final diagnosis done post-mortem
Feasibility of obtaining transgenics with cognitive deficits	They do not display neuronal loss; therefore, they are not good for testing neuroprotection in AD	Cerebrospinal fluid available	Lack of biomarkers for patient stratification (AD surely includes different underlying pathologies)
Transgenics are good models for early-onset AD (due to mutations in genes for presenilins or APP)		Pilot clinical trials are feasible. Indeed, one may wonder why there is more money allocated to studies in mice than in clinical trials	Lack of enough biomarkers to assess efficacy of intervention in clinical trials
Transgenics are good for testing cognitive enhancement strategies			Limited availability of patients, specially difficult is to obtain an homogeneous patient population
Interventions may start before pathology appears			Interventions (in clinical trials) start after diagnosis; obviously treatments cannot start before clinical symptoms appear
Animals may be simultaneously treated and trained to learn (for instance in the Morris water maze test)		**Clinical trials do not include simultaneous training to learn (interventions in animal AD models do include training)**	**Training/cognitive tasks can be included along therapeutical interventions**
Many interventions provide positive results			Many interventions in clinical trials provide inconclusive/negative results
	Many interventions lack the pharmacokinetics of the drug and data on brain penetrance		
	CSF difficult to take	CSF available	


*The table is constructed thinking of pros and cons in interventions involving animals and humans. In bold: promising strategies albeit, to our knowledge, not exploited in clinical trials.*

It is likely that the use of simple animal models reflecting a single aspect of AD is not enough to mimic the disease, and thus, to develop new treatments. Another possibility is that Aβ and tau pathologies are endpoints for different disease-driving mechanisms. Thus, achieving a successful inhibition of Aβ and tau pathologies may not result in a successful anti-AD therapy. Considering the heterogeneity of AD, it is probable that a multitarget approach will be necessary. To create better animal models for AD, an advancement in the understanding of the molecular neuropathological mechanisms would be desirable. Another key issue is the lack of neuronal death in the main animal AD models, which contrasts with the patient’s decline in CNS neuronal number. Further research breakthroughs are needed for the development of models reflecting the heterogeneity of the disease. Also, new biomakers to identify disease populations and disease progression are crucial to adjust clinical trials, and hence, evaluate the real potential of tested compounds. Yet, there is chance to find hidden clues in the huge amount of information obtained in animal models.

## LESSONS FROM PROSPECTIVE STUDIES: NEUROPROTECTION BEFORE THE DISEASE

Clinical trials for neuroprotection are extremely difficult to implement and novel strategies are being sought to be able to detect any slowing-down of disease progression. This means that, as of today, clinical trials with patients with dementia should concentrate on symptom improvement irrespective of whether it comes from neuroprotection or from enhancing cognition. Epidemiological studies are important as they provide information about neuroprotection before the disease appears or, in other words, what life style, diet habit, or chronic drug treatment may delay the onset of clinical symptoms of cognitive impairment (see the previous section). Freedman et al. (2012) reported the results of the follow-up of 5,148,760 person-years between 1995 and 2008, in which a total of 33,731 men and 18,784 women died. The conclusion of this large, prospective study is that coffee consumption inversely associates with total and cause-specific mortality. Coffee consumption may be relevant for neurodegenerative diseases for its caffeine content and its diverse and (often) positive central actions.

A relatively recent study shows in *Drosophila* models of Parkinson’s disease (PD) that decaffeinated coffee and nicotine-free tobacco provide neuroprotection (Trinh et al., 2010). Again, these animal models do not seem appropriate to extrapolate the results to humans. In fact, it has been shown that humans taking nicotine and caffeine are less prone to have PD. Ascherio et al. (2001) working with data from the Health Professionals’ Follow-Up Study and the Nurses’ Health Study cohorts (47,351 men and 88,565 women) identified a protective effect of moderate doses of caffeine on risk of PD. In parallel, data analyzed by Ross et al. (2000) from 30 years of follow-up of 8004 Japanese–American men (aged 45–68 years), enrolled in the prospective, longitudinal Honolulu Heart Program between 1965 and 1968, showed that higher coffee intake is associated with a significantly lower incidence of PD. The study of environmental, life style, and physical precursors of clinical PD from the Honolulu-Asia Aging Study indicates that factors showing an inverse association with PD included coffee intake and cigarette smoking (Abbott et al., 2003).

As one may imagine, caffeine, which is the world most consumed psychoactive molecule, was considered as a candidate to either increase or decrease the chances of having dementia. Maia and de Mendonça (2002) followed 59 AD patients and 59 controls to find using a logistic regression model, that caffeine intake in the previous 20 years was associated with a significantly lower risk for AD. The inverse correlation was confirmed by Eskelinen and Kivipelto (2010) by following for more than 20 years participants of the Cardiovascular Risk Factors, Aging and Dementia (CAIDE) study (61 cases were identified as demented – 48 having AD). The study demonstrates that coffee drinkers had lower risk of dementia and AD compared with those drinking no or only little coffee (data were indeed adjusted for depressive symptoms, apoE 𝜖4 allele and for demographic, vascular, and lifestyle factors). The lowest risk (65% decrease) was found in people who drank 3–5 cups per day. A very relevant conclusion of this study is that coffee drinking at midlife is associated with a decreased risk of dementia/AD later in life (Eskelinen and Kivipelto, 2010). Therefore, it seems that caffeine could be neuroprotective and that this could result in preserved cognition. Would the situation be similar once dementia has started?

## NEUROPROTECTION VERSUS COGNITIVE ENHANCEMENT

Protecting neurons implies not only to avoid cell death but also to preserve a functional synaptic network; therefore, the term “neuroprotection” should be extensive to both avoid neuronal and synaptic losses. Thus, a relevant question may be formulated as “what comes first”: is cognitive enhancement preventing neurodegeneration (either spine or neuron loss) or is neuroprotection that helps in retaining/retrieving memories? To answer this question, approaches to uncouple neuroprotection from cognitive enhancement would be necessary. As an analogy, understanding mitochondrial function had to await the drugs that uncoupled oxygen consumption from ATP synthesis. While oxygen consumption leads to ATP replenishment two different processes occur, namely reduction of oxygen with proton pumping across the mitochondrial membranes and oxidative phosphorylation of ADP to ATP. Uncouplers and the Nobel Laureate (in Chemistry in 1978) Peter D. Mitchell were the clue to solve the conundrum of mitochondrial operation in electron transport and synthesis of ATP ^2^.

In the AD field, accumulating reports show beneficial effects of multiple compounds on cognition in animal models. However, since these AD models do not show neuronal loss, it is expected that the reported effects on cognition result from improving neuronal performance. In humans, AD is characterized by a substantial neuronal loss, resulting in reduced brain volume; thus it is unclear that the neurodegenerative processes occurring in AD are comparable to those obtained in AD animal models. To evaluate the proper effect of drugs on neuroprotection, it would be necessary to dissect out effects on neuronal death from those on cognition.

## LESSONS FROM EFFICACIOUS AND NON-EFFICACIOUS MOLECULES IN RED WINE

Life style surely impacts on the incidence of a variety of diseases. Mediterranean diet is considered the best ally to delay or prevent the occurrence of diabetes, hypertension, obesity, etc. One key component of the diet is moderate wine consumption and resveratrol has been identified as a key compound for wine-related health benefits. For these reasons, wine and resveratrol have been considered as anti-AD agents.

Resveratrol is derived from plants and is found in highest levels in red wine and the skin of red grapes. The Copenhagen City Heart Study (Truelsen et al., 2002) reported that monthly and weekly consumption of red wine is associated with a lower risk of dementia. Subsequently, clinical studies of resveratrol are undertaken to evaluate the safety, tolerability, and effectiveness of resveratrol on CSF and other biomarkers for the treatment of AD. To give an example, the title of the NCT01504854 study is: “*Phase II Study to Evaluate the Impact on Biomarkers of Resveratrol Treatment in Patients with Mild to Moderate AD*.” Hopeful as it is, such studies with resveratrol are undermined by other clinical studies to assess the usefulness of resveratrol for (): enhancing oocyte quality in *in vitro* fertilization procedures; type II diabetes, obesity, non-alcoholic fatty liver disease, impaired glucose tolerance, insulin sensitivity and preservation of beta cell function following gestational diabetes, Friedreich’s ataxia, primary and secondary prevention of cardiovascular disease, vascular health in postmenopausal women and colon cancer. Either these ailments have a common factor or resveratrol is a miracle compound. Still, efficacy of resveratrol in AD enters into the rule that almost any drug is efficacious in AD mouse models. But are there other anti-AD ingredients in red wine?

Ho et al. (2013), in a recent and exhaustive study, measured the accumulation of polyphenols in the rat brain following oral dosage with a Cabernet Sauvignon red wine and tested brain-targeted polyphenols for potential beneficial AD disease-modifying activities. They observed that, among brain-targeted polyphenol metabolites, quercetin-3-*O*-glucuronide, was able to reduce the generation of Aβ peptides by primary neuronal cultures from the Tg2576 AD mouse model. Interestingly, another polyphenol compound, malvidin-3-*O*-glucoside, did not have an effect and therefore constitutes one of the few examples of reported negative results. The quercetin conjugate also improved AD-type deficits in hippocampal basal synaptic transmission and long-term potentiation. The authors propose that quercetin-3-*O*-glucuronide: “*may simultaneously modulate multiple independent AD disease-modifying mechanisms and, as such, may contribute to the benefits of dietary supplementation with red wines as an effective intervention for AD.*” Intriguingly, the same laboratory reported that different wines with different polyphenolic composition significantly attenuated the development of AD-type brain pathology and memory deterioration in a transgenic AD mouse model (Ho et al., 2009), postulating the possibility to develop a “*combination of dietary polyphenolic compounds for AD prevention and/or therapy by modulating multiple Abeta-related mechanisms*.” Actually, this leads us back to square one, i.e., different wines, different diet supplements, different drugs, etc., are all efficacious as anti-AD agents in animal models. The novelty is that the data in this laboratory point toward different mechanisms that may be independently (perhaps synergistically?) helpful.

## LESSONS FROM ADRENERGIC-RECEPTOR-BASED INTERVENTIONS

Some lessons could be learned from studies pointing to adrenergic receptors as targets in cognitive disorders. Whereas positive results have been described in animal models, adrenergic drugs have not reached the market for combating AD. However, a neuroprotective role for these compounds cannot be ruled out.

Adrenergic receptors are by and large the most studied G-protein-coupled receptors (GPCRs). β-Adrenergic receptor blockers and antagonists of histamine receptors started a revolution ending up with more than 40% of the current approved drugs targeting GPCRs. Among those, a huge majority target adrenergic receptors (despite there are hundreds of GPCRs). Drugs targeting those receptors arrived first to patients with cardiovascular diseases. Later on, they were approved for other indications. Although the drug is not used today for this indication, propranolol, a prototypic adrenergic receptor antagonist, was suggested for the control of disruptive behavior in dementia (Weiler et al., 1988). In the 1990s, Cowburn et al. (1993) reported disrupted β1-adrenoceptor-G protein-coupling in the temporal cortex of AD patients. Adrenoceptors and other GPCRs are deficiently coupled to G proteins in brain samples from AD patients (Wang and Friedman, 1994). Upon work with animal (rodent) models, a multivariate link between adrenoceptors and AD pathological hallmarks was established. Whereas Lee et al. (1997) reported stimulation of amyloid precursor protein synthesis by adrenergic receptors coupled to cAMP formation, Ni et al. (2006) showed that activation of β2-adrenergic receptor accelerates amyloid plaque formation and enhances γ-secretase activity.

Yu et al. (2010) reported that blocking β2-adrenergic receptor attenuates acute stress-induced Aβ peptide production, and Gibbs et al. (2010) that memory loss caused by β-amyloid protein is rescued by a β(3)-adrenoceptor agonist. Koutroumani et al. (2013) recently indicated that the deletion variant of α2b-adrenergic receptor is associated with decreased risk in AD and mild cognitive impairment. Moreover, β2 adrenergic receptor, protein kinase A (PKA) and c-Jun N-terminal kinase (JNK) signaling pathways mediate tau pathology in AD models (Wang et al., 2013). Thathiah et al. (2013) have even linked β-arrestin 2 to Aβ generation and γ-secretase activity in an animal model for AD. In terms of therapy, Katsouri et al. (2013) have tested prazosin, an α(1)-adrenoceptor antagonist, to prevent memory deterioration in the APP23 transgenic mouse model of AD. Aβ(1-42) induces internalization and degradation of β2 adrenergic receptors in prefrontal cortical neurons (Wang et al., 2011). Furthermore, it has been hypothesized that elevated norepinephrine may be an etiological factor in some cases of AD (Fitzgerald, 2010) and Bullido et al. (2004) described polymorphism in genes involved in adrenergic signaling associated with AD. Recently, Femminella et al. (2013) have written an article whose title is very suggestive: “β-adrenergic receptors and G protein-coupled receptor kinase-2 in Alzheimer’s disease: a new paradigm for prognosis and therapy?,” as suggestive is part of the summary of the Yu et al. (2010) review: “*In the recent years, several unexpected longitudinal and cross-sectional epidemiological studies reveal that beta-blocker treatment reduces the prevalence of AD in patients suffering from hypertension. Now, a newly population-based study of individuals with incident AD demonstrates that beta-blockers are also associated with delay of functional decline. Furthermore, accumulated convincing evidences from cell culture experiments and animal studies have also suggested that β-adrenergic receptors (β-ARs) may involve in the AD pathogenesis through effects on amyloid-β (Aβ) production or inflammation*.”

The above-indicated and optimistic view that adrenergic receptors as targets in AD should be taken with caution. In fact, Gliebus and Lippa (2007) highlighted that signaling via β1-adrenergic receptors is important for the retrieval of spatial and contextual memories. The main adrenergic nucleus of the brain, the* locus coeruleus* is activated by environmental cues leading to norepinephrine-mediated hippocampal activation, which is important for retrieving memories. The authors demonstrated that although β-blockers do not impair cognition in normal subjects, there was a trend for worse delayed memory retrieval in cognitively impaired patients who were on CNS-active β-blockers. The authors concluded: “*common medications used in cognitively impaired elderly patients can worsen cognition and that careful selection of medications may help to maximize retrieval of newly formed memories*.” It may happen that a given adrenergic receptor antagonist is good for neuroprotection before AD appears but not to improve patient’s condition. This hypothesis would be consistent with differential mechanism for neuroprotection and for cognitive enhancement. Moreover, two time-dependent neuroprotective strategies may be considered: one to prevent disease and another to prevent disease progression; indeed these two strategies would likely be achieved by hitting different targets. Thus, caffeine and β-blockers could delay the appearance of cognitive deficits but they may not be good for patients. In fact, caffeinated coffee is not recommended once AD has appeared.

## PHARMACOKINETICS AND MULTIMODAL DRUGS SHOULD BE TAKEN INTO ACCOUNT

The majority of studies on potential anti-AD therapies are performed on transgenic models to which the assayed substance(s) is(are) administered. In a high percentage of these studies (i) the actual concentration that reaches the CNS, (ii) the plasma/brain ratio, and (iii) the half-life of the compound in blood or brain are undetermined. These parameters are important to understand where, when, and how a given drug is acting.

It may also occur that a given drug is acting on quite diverse targets whose distribution in the (rodent or human) body is un-even. For example, Okun et al. (2010) reported a very informative study on dimebon, originally developed as an anti-histamine drug. Dimebon seems to be a multimodal drug with many different targets including serotonergic, α-adrenergic, and dopaminergic receptors. The results indicate something that should be considered in drug discovery, namely that a drug with multiple targets may result in greater benefits than a “clean” drug acting only on a given target. Furthermore, Okun et al. (2010) suggest that it is necessary to understand the role of different pathways in AD and in any other disease with complex etiologies. Such knowledge would surely help in developing multitarget drugs.

## CRITICAL ANALYSIS OF ANTI-AD THERAPEUTIC SUCCESS IN AD MOUSE MODELS

In this article, we aimed at discussing how come such diversity of targets achieves anti-AD benefits in pre-clinical studies. A common mechanism of action mediated by all these targets is difficult to envisage, and therefore it seems that different mechanisms may result in similar benefits. Very often, the benefits reported are multiple and point to the same direction. Frequently, a given drug/treatment improves cognition performance in one or various cognition tests (or strengthen long-term potentiation) and produces benefits in parameters related with one of the two main hallmarks of the disease: reduction of Aβ-containing plaques or reduction of hyperphosphorylated tau. *A priori* there is not any reason to expect that something that decreases plaques also decreases aberrant tau phosphorylation (or vice versa). Since no obvious relation has been provided to link these two pathologies (plaques and tangles) in animal models, no final conclusions may be obtained. It would be very important to know, for example, if a given drug that enhances cognition and reduces plaque load in APP mice, also shows positive effects on cognition (or even in tau pathology) in a tau mice. Even negative results would be informative. For example, which drugs/treatments do not enhance cognition but decrease amyloid burden or phosphorylated tau or which type of drug reduces one of these pathologies but worsen cognitive status.

The growing list of effective compounds curing AD mice models has only created frustration when evaluated in human trials. As to date, good animal models for AD are lacking and more comprehensive and critical studies on the results with the available models would be necessary to build high-quality knowledge.

## THEMES FOR DEBATE

The success of anti-AD interventions in mouse models together with the negative results in humans underline the gap between our models of investigation and the real disease. Models to develop a single AD pathological feature are incomplete (**Table 2**). Accelerated AD mouse models that have plaques and cognition deficits sooner in time without neuronal loss are not necessarily better models. Is it possible to create a better AD mouse model? How could we obtain the most possible information from the available models? A list of challenges is included in **Figure 2**.

Actions leading to cognitive enhancement even in AD models may be considered for use in humans, especially in cases in which the drug is already in the market (drug reprofiling programs). In addition of reducing amyloid and tau pathologies, would it be necessary to boost synaptic strength and cognition?

Common mechanisms in FAD and late-onset AD are not evident. Is that anti-amyloid strategies would have limited benefits only in FAD cases?

Many diverse genetic and environmental factors have been shown to contribute to AD. This could imply that the etiology of AD is multiple. The field is in need of biomarkers to refine criteria for achieving a better patient stratification. Are we targeting the right population in clinical trials?

Retrospective epidemiological studies have provided evidence for good AD preventive compounds. Some of them were tried in humans with negative results. Would it be convenient to investigate the putative effects of these compounds in prospective studies?

### CHALLENGES

The discovery of genetic and environmental risk factors for AD offers the possibility of designing preventing strategies, but also of understanding how the disease starts and thus, of finding novel disease-modifying therapies. It is still questionable if anti-Aβ-based therapies will provide the expected benefits. Perhaps, anti-Aβ approaches would only work for FAD cases, where the amyloid pathogenesis is prominent. Anti-tau-based therapies have also failed in clinical trials, thus raising the possibility that anti-tau interventions come too late to prevent neurodegeneration in AD. One of the biggest challenges for AD research is to create good animal models that adequately reflect both disease etiology and disease progression. Accumulating evidence demonstrate that the current models, based in massive overexpression of Aβ or p-tau, are not fit for purpose. Since AD etiology seems to be heterogeneous (several variant genetic and lifestyle risk factors reported), it would be instrumental to have specific biomarkers for each subgroup of patients. Advances in all those aspects will bring the possibility of personalized preventive and curative strategies.

## Conflict of Interest Statement

The authors declare that the research was conducted in the absence of any commercial or financial relationships that could be construed as a potential conflict of interest.

## Footnotes

1 Trisomy in the chromosome 21, where the APP gene is also present.

2 Some scientists are reluctant to accept Mitchell’s chemiosmotic hypothesis; however to date it is the one that best describes the phenomenon.

## References

[B1] Abbott R. D. Ross G. W. White L. R. Sanderson W. T. Burchfiel C. M. Kashon M. (2003). Environmental, life-style, and physical precursors of clinical Parkinson’s disease: recent findings from the Honolulu-Asia Aging Study. *J. Neurol.* 250(Suppl. 3) III30–III39 10.1007/s00415-003-1306-7 14579122

[B2] Ascherio A. Zhang S. M. Hernan M. A. Kawachi I. Colditz G. A. Speizer F. E. (2001). Prospective study of caffeine consumption and risk of Parkinson’s disease in men and women. *Ann. Neurol.* 50 56–63 10.1002/ana.1052 11456310

[B3] Aydin D. Weyer S. W. Muller U. C. (2012). Functions of the APP gene family in the nervous system: insights from mouse models. *Exp. Brain Res.* 217 423–434 10.1007/s00221-011-2861-2 21931985

[B4] Bales K. R. Verina T. Cummins D. J. Du Y. Dodel R. C. Saura J. (1999). Apolipoprotein E is essential for amyloid deposition in the APP(V717F) transgenic mouse model of Alzheimer’s disease. *Proc. Natl. Acad. Sci. U.S.A.* 96 15233–15238 10.1073/pnas.96.26.15233 10611368 PMC24803

[B5] Barthet G. Georgakopoulos A. Robakis N. K. (2012). Cellular mechanisms of gamma-secretase substrate selection, processing and toxicity. *Prog. Neurobiol.* 98 166–175 10.1016/j.pneurobio.2012.05.006 22622135 PMC3404154

[B6] Brunden K. R. Zhang B. Carroll J. Yao Y. Potuzak J. S. Hogan A. M. (2010). Epothilone D improves microtubule density, axonal integrity, and cognition in a transgenic mouse model of tauopathy. *J. Neurosci.* 30 13861–13866 10.1523/JNEUROSCI.3059-10.2010 20943926 PMC2958430

[B7] Bullido M. J. Ramos M. C. Ruiz-Gomez A. Tutor A. S. Sastre I. Frank A. (2004). Polymorphism in genes involved in adrenergic signaling associated with Alzheimer’s. *Neurobiol. Aging* 25 853–859 10.1016/j.neurobiolaging.2003.10.006 15212839

[B8] Calzà L. Baldassarro V. A. Giuliani A. Lorenzini L. Fernandez M. Mangano C. (2013). From the multifactorial nature of Alzheimer’s disease to multitarget therapy: the contribution of the translational approach. *Curr. Top. Med. Chem.* 13 1843–1852 10.2174/15680266113139990140 23931439

[B9] Carvajal F. J. Zolezzi J. M. Tapia-Rojas C. Godoy J. A. Inestrosa N. C. (2013). Tetrahydrohyperforin decreases cholinergic markers associated with amyloid-beta plaques, 4-hydroxynonenal formation, and caspase-3 activation in AbetaPP/PS1 mice. *J. Alzheimers Dis.* 36 99–118 23568104 10.3233/JAD-130230

[B10] Castellani R. J. Smith M. A. (2011). Compounding artefacts with uncertainty, and an amyloid cascade hypothesis that is ‘too big to fail’. *J. Pathol*. 224 147–152 10.1002/path.2885 21557219

[B11] Cedazo-Minguez A. Cowburn R. F. (2001). Apolipoprotein E isoform-specific disruption of phosphoinositide hydrolysis: protection by estrogen and glutathione. *FEBS Lett.* 504 45–49 10.1016/S0014-5793(01)02761-2 11522294

[B12] Cedazo-Minguez A. Huttinger M. Cowburn R. F. (2001). Beta-VLDL protects against A beta(1-42) and apoE toxicity in human SH-SY5Y neuroblastoma cells. *Neuroreport* 12 201–206 10.1097/00001756-200102120-00006 11209921

[B13] Cheng X. S. Zhao K. P. Jiang X. Du L. L. Li X. H. Ma Z. W. (2013). Nmnat2 attenuates Tau phosphorylation through activation of PP2A. *J. Alzheimers Dis.* 36 185–195 10.3233/JAD-122173 23579329

[B14] Cleary J. P. Walsh D. M. Hofmeister J. J. Shankar G. M. Kuskowski M. A. Selkoe D. J. (2005). Natural oligomers of the amyloid-beta protein specifically disrupt cognitive function. *Nat. Neurosci.* 8 79–84 10.1038/nn1372 15608634

[B15] Congdon E. E. Wu J. W. Myeku N. Figueroa Y. H. Herman M. Marinec P. S. (2012). Methylthioninium chloride (methylene blue) induces autophagy and attenuates tauopathy in vitro and in vivo. *Autophagy* 8 609–622 10.4161/auto.19048 22361619 PMC3405840

[B16] Corcoran N. M. Martin D. Hutter-Paier B. Windisch M. Nguyen T. Nheu L. (2010). Sodium selenate specifically activates PP2A phosphatase, dephosphorylates tau and reverses memory deficits in an Alzheimer’s disease model. *J. Clin. Neurosci.* 17 1025–1033 10.1016/j.jocn.2010.04.020 20537899

[B17] Corder E. H. Saunders A. M. Strittmatter W. J. Schmechel D. E. Gaskell P. C. Small G. W. (1993). Gene dose of apolipoprotein E type 4 allele and the risk of Alzheimer’s disease in late onset families. *Science* 261 921–923 10.1126/science.8346443 8346443

[B18] Cowburn R. F. Popescu B. O. Ankarcrona M. Dehvari N. Cedazo-Minguez A. (2007). Presenilin-mediated signal transduction. *Physiol. Behav.* 92 93–97 10.1016/j.physbeh.2007.05.053 17568632

[B19] Cowburn R. F. Vestling M. Fowler C. J. Ravid R. Winblad B. O’Neill C. (1993). Disrupted beta 1-adrenoceptor-G protein coupling in the temporal cortex of patients with Alzheimer’s disease. *Neurosci. Lett.* 155 163–166 10.1016/0304-3940(93)90698-K 8397350

[B20] Cuadrado-Tejedor M. García-Osta A. Ricobaraza A. Oyarzabal J. Franco R. (2011). Defining the mechanism of action of 4-phenylbutyrate to develop a small-molecule-based therapy for Alzheimer’s disease. *Curr. Med. Chem.* 18 5545–5553 10.2174/092986711798347315 22172064

[B21] Davinelli S. Sapere N. Zella D. Bracale R. Intrieri M. Scapagnini G. (2012). Pleiotropic protective effects of phytochemicals in Alzheimer’s disease. *Oxid. Med. Cell Longev*. 2012:386527 10.1155/2012/386527 PMC3368517 22690271

[B22] De la Monte S. M. Tong M. (2014). Brain metabolic dysfunction at the core of Alzheimer’s disease. *Biochem. Pharmacol.* 88 548–559 10.1016/j.bcp.2013.12.012 24380887 PMC4550323

[B23] Dinamarca M. C. Cerpa W. Garrido J. Hancke J. L. Inestrosa N. C. (2006). Hyperforin prevents beta-amyloid neurotoxicity and spatial memory impairments by disaggregation of Alzheimer’s amyloid-beta-deposits. *Mol. Psychiatry* 11 1032–1048 10.1038/sj.mp.4001866 16880827

[B24] Duce J. A. Tsatsanis A. Cater M. A. James S. A. Robb E. Wikhe K. (2010). Iron-export ferroxidase activity of beta-amyloid precursor protein is inhibited by zinc in Alzheimer’s disease. *Cell* 142 857–867 10.1016/j.cell.2010.08.014 20817278 PMC2943017

[B25] Dursun E. Gezen-Ak D. Yilmazer S. (2013). A new mechanism for amyloid-beta induction of iNOS: vitamin D-VDR pathway disruption. *J. Alzheimers Dis.* 36 459–474 10.3233/JAD-130416 23624519

[B26] Ebneth A. Godemann R. Stamer K. Illenberger S. Trinczek B. Mandelkow E. (1998). Overexpression of tau protein inhibits kinesin-dependent trafficking of vesicles, mitochondria, and endoplasmic reticulum: implications for Alzheimer’s disease. *J. Cell Biol.* 143 777–794 10.1083/jcb.143.3.777 9813097 PMC2148132

[B27] Engelborghs S. Gilles C. Ivanoiu A. Vandewoude M. (2014). Rationale and clinical data supporting nutritional intervention in Alzheimer’s disease. *Acta Clin. Belg.* 69 17–24 10.1179/0001551213Z.0000000006 24635394

[B28] Eskelinen M. H. Kivipelto M. (2010). Caffeine as a protective factor in dementia and Alzheimer’s disease. *J. Alzheimers Dis.* 20(Suppl. 1) S167–S174 10.3233/JAD-2010-1404 20182054

[B29] Femminella G. D. Rengo G. Pagano G. de Lucia C. Komici K. Parisi V. (2013). Beta-adrenergic receptors and G protein-coupled receptor kinase-2 in Alzheimer’s disease: a new paradigm for prognosis and therapy? *J. Alzheimers Dis.* 34 341–347 10.3233/JAD-121813 23207488

[B30] Ferreira S. T. Clarke J. R. Bomfim T. R. De Felice F. G. (2014). Inflammation, defective insulin signaling, and neuronal dysfunction in Alzheimer’s disease. *Alzheimers Dement.* 10 S76–S83 10.1016/j.jalz.2013.12.010 24529528

[B31] Fitzgerald P. J. (2010). Is elevated norepinephrine an etiological factor in some cases of Alzheimer’s disease? *Curr. Alzheimer Res.* 7 506–516 10.2174/156720510792231775 20626335

[B32] Fox M. Berzuini C. Knapp L. A. (2013). Maternal breastfeeding history and Alzheimer’s disease risk. *J. Alzheimers Dis.* 37 809–821 10.3233/JAD-130152 23948914

[B33] Freedman N. D. Park Y. Abnet C. C. Hollenbeck A. R. Sinha R. (2012). Association of coffee drinking with total and cause-specific mortality. *N. Engl. J. Med.* 366 1891–1904 10.1056/NEJMoa1112010 22591295 PMC3439152

[B34] Fuentes P. Catalan J. A. (2011). Clinical perspective: anti tau’s treatment in Alzheimer’s disease. *Curr. Alzheimer Res.* 8 686–688 10.2174/156720511796717221 21605037

[B35] García-Osta A. Cuadrado-Tejedor M. García-Barroso C. Oyarzábal J. Franco R. (2012). Phosphodiesterases as therapeutic targets for Alzheimer’s disease. *ACS Chem. Neurosci.* 3 832–844 10.1021/cn3000907 23173065 PMC3503343

[B36] Gersbacher M. T. Kim D. Y. Bhattacharyya R. Kovacs D. M. (2010). Identification of BACE1 cleavage sites in human voltage-gated sodium channel beta 2 subunit. *Mol. Neurodegener.* 5:61 10.1186/1750-1326-5-61 PMC3022600 21182789

[B37] Gibbs M. E. Maksel D. Gibbs Z. Hou X. Summers R. J. Small D. H. (2010). Memory loss caused by beta-amyloid protein is rescued by a beta(3)-adrenoceptor agonist. *Neurobiol. Aging* 31 614–624 10.1016/j.neurobiolaging.2008.05.018 18632189

[B38] Gliebus G. Lippa C. F. (2007). The influence of beta-blockers on delayed memory function in people with cognitive impairment. *Am. J. Alzheimers Dis. Other Demen.* 22 57–61 10.1177/1533317506295889 17534003 PMC10697206

[B39] Goedert M. Jakes R. (2005). Mutations causing neurodegenerative tauopathies. *Biochim. Biophys. Acta* 1739 240–250 10.1016/j.bbadis.2004.08.007 15615642

[B40] Gong C. X. Liu F. Grundke-Iqbal I. Iqbal K. (2006). Impaired brain glucose metabolism leads to Alzheimer neurofibrillary degeneration through a decrease in tau O-GlcNAcylation. *J. Alzheimer’s Dis.* 9 1–12 16627930 10.3233/jad-2006-9101

[B41] Griffith T. N. Varela-Nallar L. Dinamarca M. C. Inestrosa N. C. (2010). Neurobiological effects of hyperforin and its potential in Alzheimer’s disease therapy. *Curr. Med. Chem.* 17 391–406 10.2174/092986710790226156 20015041

[B42] Haapasalo A. Kovacs D. M. (2011). The many substrates of presenilin/gamma-secretase. *J. Alzheimer’s Dis.* 25 3–28 10.3233/JAD-2011-101065 21335653 PMC3281584

[B43] Hardy J. A. Higgins G. A. (1992). Alzheimer’s disease: the amyloid cascade hypothesis. *Science* 256 184–185 10.1126/science.1566067 1566067

[B44] Hardy J. Bogdanovic N. Winblad B. Portelius E. Andreasen N. Cedazo-Minguez A. (2014). Pathways to Alzheimer’s disease. *J. Intern. Med.* 275 296–303 10.1111/joim.12192 24749173

[B45] Ho L. Chen L. H. Wang J. Zhao W. Talcott S. T. Ono K. (2009). Heterogeneity in red wine polyphenolic contents differentially influences Alzheimer’s disease-type neuropathology and cognitive deterioration. *J. Alzheimers Dis.* 16 59–72 10.3233/JAD-2009-0916 19158422 PMC2857553

[B46] Ho L. Ferruzzi M. G. Janle E. M. Wang J. Gong B. Chen T. Y. (2013). Identification of brain-targeted bioactive dietary quercetin-3-*O*-glucuronide as a novel intervention for Alzheimer’s disease. *FASEB J.* 27 769–781 10.1096/fj.12-212118 23097297 PMC3545533

[B47] Hu X. Hicks C. W. He W. Wong P. Macklin W. B. Trapp B. D. (2006). Bace1 modulates myelination in the central and peripheral nervous system. *Nat. Neurosci.* 9 1520–1525 10.1038/nn1797 17099708

[B48] Imtiaz B. Tolppanen A. M. Kivipelto M. Soininen H. (2014). Future directions in Alzheimer’s disease from risk factors to prevention. *Biochem. Pharmacol.* 88 661–670 10.1016/j.bcp.2014.01.003 24418410

[B49] Jurisch-Yaksi N. Sannerud R. Annaert W. (2013). A fast growing spectrum of biological functions of gamma-secretase in development and disease. *Biochim. Biophys. Acta* 1828 2815–2827 10.1016/j.bbamem.2013.04.016 24099003

[B50] Katsouri L. Vizcaychipi M. P. McArthur S. Harrison I. Suárez-Calvet M. Lleo A. (2013). Prazosin, an α(1)-adrenoceptor antagonist, prevents memory deterioration in the APP23 transgenic mouse model of Alzheimer’s disease. *Neurobiol. Aging* 34 1105–1115 10.1016/j.neurobiolaging.2012.09.010 23063647

[B51] Kivipelto M. Rovio S. Ngandu T. Kareholt I. Eskelinen M. Winblad B. (2008). Apolipoprotein E epsilon4 magnifies lifestyle risks for dementia: a population-based study. *J. Cell. Mol. Med.* 12 2762–2771 10.1111/j.1582-4934.2008.00296.x 18318693 PMC3828889

[B52] Klemow K. M. Bartlow A. Crawford J. Kocher N. Shah J. Ritsick M. (2011). “Medical Attributes of St. John’s Wort (*Hypericum perforatum*),” in *Herbal Medicine: Biomolecular and Clinical Aspects* 2nd Edn eds Benzie I. F. F. Wachtel-Galor S. (Boca Raton, FL: CRC Press). 22593920

[B53] Koutroumani M. Daniilidou M. Giannakouros T. Proitsi P. Liapi D. Germanou A. (2013). The deletion variant of alpha2b-adrenergic receptor is associated with decreased risk in Alzheimer’s disease and mild cognitive impairment. *J. Neurol. Sci.* 328 19–23 10.1016/j.jns.2013.02.003 23499426

[B54] LaDu M. J. Falduto M. T. Manelli A. M. Reardon C. A. Getz G. S. Frail D. E. (1994). Isoform-specific binding of apolipoprotein E to beta-amyloid. *J. Biol. Chem.* 269 23403–23406 8089103

[B55] Lambert M. P. Barlow A. K. Chromy B. A. Edwards C. Freed R. Liosatos M. (1998). Diffusible, nonfibrillar ligands derived from Abeta1-42 are potent central nervous system neurotoxins. *Proc. Natl. Acad. Sci. U.S.A.* 95 6448–6453 10.1073/pnas.95.11.6448 9600986 PMC27787

[B56] Lee H. G. Casadesus G. Zhu X. Takeda A. Perry G. Smith M. A. (2004). Challenging the amyloid cascade hypothesis: senile plaques and amyloid-beta as protective adaptations to Alzheimer disease. *Ann. N. Y. Acad. Sci.* 1019 1–4 10.1196/annals.1297.001 15246983

[B57] Lee R. K. Araki W. Wurtman R. J. (1997). Stimulation of amyloid precursor protein synthesis by adrenergic receptors coupled to cAMP formation. *Proc. Natl. Acad. Sci. U.S.A.* 94 5422–5426 10.1073/pnas.94.10.5422 9144253 PMC24694

[B58] Ligthart S. A. Moll van Charante E. P. Van Gool W. A. Richard E. (2010). Treatment of cardiovascular risk factors to prevent cognitive decline and dementia: a systematic review. *Vasc. Health Risk Manag.* 6 775–785 10.2147/VHRM.S7343 20859546 PMC2941788

[B59] Lorenzo A. Yankner B. A. (1994). Beta-amyloid neurotoxicity requires fibril formation and is inhibited by congo red. *Proc. Natl. Acad. Sci. U.S.A.* 91 12243–12247 10.1073/pnas.91.25.12243 7991613 PMC45413

[B60] Maia L. de Mendonça A. (2002). Does caffeine intake protect from Alzheimer’s disease? *Eur. J. Neurol.* 9 377–382 10.1046/j.1468-1331.2002.00421.x 12099922

[B61] Manelli A. M. Bulfinch L. C. Sullivan P. M. LaDu M. J. (2007). Abeta42 neurotoxicity in primary co-cultures: effect of apoE isoform and Abeta conformation. *Neurobiol. Aging* 28 1139–1147 10.1016/j.neurobiolaging.2006.05.024 16837105 PMC3752940

[B62] Mannu P. Rinaldi S. Fontani V. Castagna A. (2011). Radio electric asymmetric brain stimulation in the treatment of behavioral and psychiatric symptoms in Alzheimer disease. *Clin. Interv. Aging* 6 207–211 21822377 10.2147/CIA.S23394 PMC3147052

[B63] Matsuoka Y. Jouroukhin Y. Gray A. J. Ma L. Hirata-Fukae C. Li H. F. (2008). A neuronal microtubule-interacting agent, NAPVSIPQ, reduces tau pathology and enhances cognitive function in a mouse model of Alzheimer’s disease. *J. Pharmacol. Exp. Ther.* 325 146–153 10.1124/jpet.107.130526 18199809

[B64] Medina D. X. Caccamo A. Oddo S. (2011). Methylene blue reduces abeta levels and rescues early cognitive deficit by increasing proteasome activity. *Brain Pathol.* 21 140–149 10.1111/j.1750-3639.2010.00430.x 20731659 PMC2992595

[B65] Medina M. Avila J. (2010). Glycogen synthase kinase-3 (GSK-3) inhibitors for the treatment of Alzheimer’s disease. *Curr. Pharm. Des.* 16 2790–2798 10.2174/138161210793176581 20698823

[B66] Medina M. Avila J. (2014). New perspectives on the role of tau in Alzheimer’s disease. Implications for therapy. *Biochem. Pharmacol.* 88. 540–547 10.1016/j.bcp.2014.01.013 24462919

[B67] Morimoto B. H. Schmechel D. Hirman J. Blackwell A. Keith J. Gold M. (2013). A double-blind, placebo-controlled, ascending-dose, randomized study to evaluate the safety, tolerability and effects on cognition of AL-108 after 12 weeks of intranasal administration in subjects with mild cognitive impairment. *Dement. Geriatr. Cogn. Disord.* 35 325–336 10.1159/000348347 23594991

[B68] Ni Y. Zhao X. Bao G. Zou L. Teng L. Wang Z. (2006). Activation of beta2-adrenergic receptor stimulates gamma-secretase activity and accelerates amyloid plaque formation. *Nat. Med.* 12 1390–1396 10.1038/nm1485 17115048

[B69] Octave J. N. Pierrot N. Ferao Santos S. Nalivaeva N. N. Turner A. J. (2013). From synaptic spines to nuclear signaling: nuclear and synaptic actions of the amyloid precursor protein. *J. Neurochem.* 126 183–190 10.1111/jnc.12239 23495999

[B70] Okun I. Tkachenko S. E. Khvat A. Mitkin O. Kazey V. Ivachtchenko A. V. (2010). From anti-allergic to anti-Alzheimer’s: molecular pharmacology of Dimebon. *Curr. Alzheimer Res.* 7 97–112 10.2174/156720510790691100 19939222

[B71] Olazarán J. Gonzalez B. Lopez-Alvarez J. Castagna A. Osa-Ruiz E. Herrero-Cano V. (2013). Motor effects of REAC in advanced Alzheimer’s disease: results from a pilot trial. *J. Alzheimers Dis.* 36 297–302 10.3233/JAD-130077 23603397

[B72] Panza F. Frisardi V. Solfrizzi V. Imbimbo B. P. Logroscino G. Santamato A. (2012). Immunotherapy for Alzheimer’s disease: from anti-beta-amyloid to tau-based immunization strategies. *Immunotherapy* 4 213–238 10.2217/imt.11.170 22339463

[B73] Pardossi-Piquard R. Checler F. (2012). The physiology of the beta-amyloid precursor protein intracellular domain AICD. *J. Neurochem.* 120(Suppl. 1) 109–124 10.1111/j.1471-4159.2011.07475.x 22122663

[B74] Qiu W. Q. Mwamburi M. Besser L. M. Zhu H. Li H. Wallack M. (2013). Angiotensin converting enzyme inhibitors and the reduced risk of Alzheimer’s disease in the absence of apolipoprotein E4 allele. *J. Alzheimers Dis.* 37 421–428 10.3233/JAD-130716 23948883 PMC3972060

[B75] Rice H. C. Townsend M. Bai J. Suth S. Cavanaugh W. Selkoe D. J. (2012). Pancortins interact with amyloid precursor protein and modulate cortical cell migration. *Development* 139 3986–3996 10.1242/dev.082909 22992957 PMC3472593

[B76] Rivest S. (2009). Regulation of innate immune responses in the brain. *Nat. Rev. Immunol.* 9 429–439 10.1038/nri2565 19461673

[B77] Rosini M. Simoni E. Milelli A. Minarini A. Melchiorre C. (2013). Oxidative stress in Alzheimer’s disease: are we connecting the dots? *J. Med. Chem.* 57 2821–2831 10.1021/jm400970m 24131448

[B78] Ross G. W. Abbott R. D. Petrovitch H. Morens D. M. Grandinetti A. Tung K. H. (2000). Association of coffee and caffeine intake with the risk of Parkinson disease. *JAMA* 283 2674–2679 10.1001/jama.283.20.2674 10819950

[B79] Ryan J. P. Fine D. F. Rosano C. (2014). Type 2 diabetes and cognitive impairment: contributions from neuroimaging. *J. Geriatr. Psychiatry Neurol.* 27 47–55 10.1177/0891988713516543 24394151 PMC4049175

[B80] Sandebring A. Welander H. Winblad B. Graff C. Tjernberg L. O. (2013). The pathogenic abeta43 is enriched in familial and sporadic Alzheimer disease. *PLoS ONE* 8:e55847 10.1371/journal.pone.0055847 PMC3569467 23409063

[B81] Saydoff J. A. Olariu A. Sheng J. Hu Z. Li Q. Garcia R. (2013). Uridine prodrug improves memory in Tg2576 and TAPP mice and reduces pathological factors associated with Alzheimer’s disease in related models. *J. Alzheimers Dis.* 36 637–657 10.3233/JAD-130059 23648515

[B82] Schmechel D. E. Saunders A. M. Strittmatter W. J. Crain B. J. Hulette C. M. Joo S. H. (1993). Increased amyloid beta-peptide deposition in cerebral cortex as a consequence of apolipoprotein E genotype in late-onset Alzheimer disease. *Proc. Natl. Acad. Sci. U.S.A.* 90 9649–9653 10.1073/pnas.90.20.9649 8415756 PMC47627

[B83] Schneider A. Mandelkow E. (2008). Tau-based treatment strategies in neurodegenerative diseases. *Neurotherapeutics* 5 443–457 10.1016/j.nurt.2008.05.006 18625456 PMC5084246

[B84] Sepehry A. A. Lee P. E. Hsiung G. Y. Beattie B. L. Jacova C. (2012). Effect of selective serotonin reuptake inhibitors in Alzheimer’s disease with comorbid depression: a meta-analysis of depression and cognitive outcomes. *Drugs Aging* 29 793–806 10.1007/s40266-012-0012-5 23079957

[B85] Shankar G. M. Li S. Mehta T. H. Garcia-Munoz A. Shepardson N. E. Smith I. (2008). Amyloid-beta protein dimers isolated directly from Alzheimer’s brains impair synaptic plasticity and memory. *Nat. Med.* 14 837–842 10.1038/nm1782 18568035 PMC2772133

[B86] Shinohara M. Sato N. Shimamura M. Kurinami H. Hamasaki T. Chatterjee A. (2014). Possible modification of Alzheimer’s disease by statins in midlife: interactions with genetic and non-genetic risk factors. *Front. Aging Neurosci*. 6:71 10.3389/fnagi.2014.00071 PMC4005936 24795626

[B87] Shively S. Scher A. I. Perl D. P. Diaz-Arrastia R. (2012). Dementia resulting from traumatic brain injury: what is the pathology? *Arch. Neurol.* 69 1245–1251 10.1001/archneurol.2011.3747 22776913 PMC3716376

[B88] Sontag E. M. Lotz G. P. Agrawal N. Tran A. Aron R. Yang G. (2012). Methylene blue modulates huntingtin aggregation intermediates and is protective in Huntington’s disease models. *J. Neurosci.* 32 11109–11119 10.1523/JNEUROSCI.0895-12.2012 22875942 PMC3546821

[B89] Soscia S. J. Kirby J. E. Washicosky K. J. Tucker S. M. Ingelsson M. Hyman B. (2010). The Alzheimer’s disease-associated amyloid beta-protein is an antimicrobial peptide. *PLoS ONE* 5:e9505 10.1371/journal.pone.0009505 PMC2831066 20209079

[B90] Tabaton M. Zhu X. Perry G. Smith M. A. Giliberto L. (2010). Signaling effect of amyloid-beta(42) on the processing of AbetaPP. *Exp. Neurol.* 221 18–25 10.1016/j.expneurol.2009.09.002 19747481 PMC2812589

[B91] Tanzi R. E. Kovacs D. M. Kim T. W. Moir R. D. Guenette S. Y. Wasco W. (1996). The gene defects responsible for familial Alzheimer’s disease. *Neurobiol. Dis.* 3 159–168 10.1006/nbdi.1996.0016 8980016

[B92] Thathiah A. Horre K. Snellinx A. Vandewyer E. Huang Y. Ciesielska M. (2013). Beta-arrestin 2 regulates Abeta generation and gamma-secretase activity in Alzheimer’s disease. *Nat. Med.* 19 43–49 10.1038/nm.3023 23202293

[B93] Toda N. Kaneko T. Kogen H. (2010). Development of an efficient therapeutic agent for Alzheimer’s disease: design and synthesis of dual inhibitors of acetylcholinesterase and serotonin transporter. *Chem. Pharm. Bull.* (*Tokyo*) 58 273–287 10.1248/cpb.58.273 20190429

[B94] Trinh K. Andrews L. Krause J. Hanak T. Lee D. Gelb M. (2010). Decaffeinated coffee and nicotine-free tobacco provide neuroprotection in Drosophila models of Parkinson’s disease through an NRF2-dependent mechanism. *J. Neurosci.* 30 5525–5532 10.1523/JNEUROSCI.4777-09.2010 20410106 PMC3842467

[B95] Truelsen T. Thudium D. Gronbaek M. (2002). Amount and type of alcohol and risk of dementia: the Copenhagen City Heart Study. *Neurology* 59 1313–1319 10.1212/01.WNL.0000031421.50369.E7 12427876

[B96] Van Bebber F. Paquet D. Hruscha A. Schmid B. Haass C. (2010). Methylene blue fails to inhibit Tau and polyglutamine protein dependent toxicity in zebrafish. *Neurobiol. Dis.* 39 265–271 10.1016/j.nbd.2010.03.023 20381619

[B97] Vestling M. Cedazo-Minguez A. Adem A. Wiehager B. Racchi M. Lannfelt L. (1999). Protein kinase C and amyloid precursor protein processing in skin fibroblasts from sporadic and familial Alzheimer’s disease cases. *Biochim. Biophys. Acta* 1453 341–350 10.1016/S0925-4439(99)00003-4 10101252

[B98] Veszelka S. Toth A. E. Walter F. R. Datki Z. Mozes E. Fulop L. (2013). Docosahexaenoic acid reduces amyloid-beta induced toxicity in cells of the neurovascular unit. *J. Alzheimers Dis.* 36 487–501 10.3233/JAD-120163 23645098

[B99] Virmani A. Pinto L. Binienda Z. Ali S. (2013). Food, nutrigenomics, and neurodegeneration – neuroprotection by what you eat! *Mol. Neurobiol.* 48 353–362 10.1007/s12035-013-8498-3 23813102

[B100] Wang D. Fu Q. Zhou Y. Xu B. Shi Q. Igwe B. (2013). Beta2 adrenergic receptor, protein kinase A (PKA) and c-Jun N-terminal kinase (JNK) signaling pathways mediate tau pathology in Alzheimer disease models. *J. Biol. Chem.* 288 10298–10307 10.1074/jbc.M112.415141 23430246 PMC3624413

[B101] Wang D. Yuen E. Y. Zhou Y. Yan Z. Xiang Y. K. (2011). Amyloid beta peptide-(1-42) induces internalization and degradation of beta2 adrenergic receptors in prefrontal cortical neurons. *J. Biol. Chem.* 286 31852–31863 10.1074/jbc.M111.244335 21757762 PMC3173113

[B102] Wang H. Y. Friedman E. (1994). Receptor-mediated activation of G proteins is reduced in postmortem brains from Alzheimer’s disease patients. *Neurosci. Lett.* 173 37–39 10.1016/0304-3940(94)90144-9 7936419

[B103] Weiler P. G. Mungas D. Bernick C. (1988). Propranolol for the control of disruptive behavior in senile dementia. *J. Geriatr. Psychiatry Neurol.* 1 226–230 10.1177/089198878800100408 3252890

[B104] Wiesmann M. Jansen D. Zerbi V. Broersen L. M. Garthe A. Kiliaan A. J. (2013a). Improved spatial learning strategy and memory in aged Alzheimer AbetaPPswe/PS1dE9 mice on a multi-nutrient diet. *J. Alzheimers Dis.* 37 233–245 10.3233/JAD-130179 23803297

[B105] Wiesmann M. Kiliaan A. J. Claassen J. A. (2013b). Vascular aspects of cognitive impairment and dementia. *J. Cereb. Blood Flow Metab*. 33 1696–1706 10.1038/jcbfm.2013.159 [Epub 2013 Sep 11] 24022624 PMC3824191

[B106] Wilcock D. M. Griffin W. S. (2013). Down’s syndrome, neuroinflammation, and Alzheimer neuropathogenesis. *J. Neuroinflammation* 10:84 10.1186/1742-2094-10-84 PMC3750399 23866266

[B107] Willem M. Garratt A. N. Novak B. Citron M. Kaufmann S. Rittger A. (2006). Control of peripheral nerve myelination by the beta-secretase BACE1. *Science* 314 664–666 10.1126/science.1132341 16990514

[B108] Yang Y. Song W. (2013). Molecular links between Alzheimer’s disease and diabetes mellitus. *Neuroscience* 250 140–150 10.1016/j.neuroscience.2013.07.009 23867771

[B109] Yao Z. X. Papadopoulos V. (2002). Function of beta-amyloid in cholesterol transport: a lead to neurotoxicity. *FASEB J.* 16 1677–1679 10.1096/fj.02-0285fje 12206998

[B110] Yu N. N. Wang X. X. Yu J. T. Wang N. D. Lu R. C. Miao D. (2010). Blocking beta2-adrenergic receptor attenuates acute stress-induced amyloid beta peptides production. *Brain Res.* 1317 305–310 10.1016/j.brainres.2009.12.087 20059989

[B111] Zhang X. Le W. (2010). Pathological role of hypoxia in Alzheimer’s disease. *Exp. Neurol.* 223 299–303 10.1016/j.expneurol.2009.07.033 19679125

[B112] Zhao L. Mao Z. Chen S. Schneider L. S. Brinton R. D. (2013). Early intervention with an estrogen receptor beta-selective phytoestrogenic formulation prolongs survival, improves spatial recognition memory, and slows progression of amyloid pathology in a female mouse model of Alzheimer’s disease. *J. Alzheimers Dis.* 37 403–419 10.3233/JAD-122341 23948892 PMC4197935

[B113] Zolezzi J. M. Carvajal F. J. Rios J. A. Ordenes D. Silva-Alvarez C. Godoy J. A. (2013). Tetrahydrohyperforin induces mitochondrial dynamics and prevents mitochondrial Ca^2+^ overload after Abeta and Abeta-AChE complex challenge in rat hippocampal neurons. *J. Alzheimers Dis.* 37 735–746 10.3233/JAD-130173 23948911

[B114] Zou K. Gong J. S. Yanagisawa K. Michikawa M. (2002). A novel function of monomeric amyloid beta-protein serving as an antioxidant molecule against metal-induced oxidative damage. *J. Neurosci.* 22 4833–4841 12077180 10.1523/JNEUROSCI.22-12-04833.2002 PMC6757724

